# Age-dependent rise in IFN-γ competence undermines effective type 2 responses to nematode infection

**DOI:** 10.1038/s41385-022-00519-6

**Published:** 2022-06-11

**Authors:** Bhavya Kapse, Hongwei Zhang, Nicole Affinass, Friederike Ebner, Susanne Hartmann, Sebastian Rausch

**Affiliations:** Institute of Immunology, Centre for Infection Medicine, Freie Universität Berlin, Berlin, Germany

## Abstract

The efficient induction of type 2 immune responses is central to the control of helminth infections. Previous studies demonstrated that strong Th1 responses driven by intracellular pathogens as well as a bias for type 1 activity in senescent mice impedes the generation of Th2 responses and the control of intestinal nematode infections. Here, we show that the spontaneous differentiation of Th1 cells and their expansion with age restrains type 2 immunity to infection with the small intestinal nematode *H. polygyrus* much earlier in life than previously anticipated. This includes the more extensive induction of IFN-γ competent, nematode-specific Th2/1 hybrid cells in BALB/c mice older than three months compared to younger animals. In C57BL/6 mice, Th1 cells accumulate more rapidly at steady state, translating to elevated Th2/1 differentiation and poor control of parasite fitness in primary infections experienced at a young age. Blocking of early IFN-γ and IL-12 signals during the first week of nematode infection leads to sharply decreased Th2/1 differentiation and promotes resistance in both mouse lines. Together, these data suggest that IFN-γ competent, type 1 like effector cells spontaneously accumulating in the vertebrate host progressively curtail the effectiveness of anti-nematode type 2 responses with rising host age.

## Introduction

Experimental work in rodent models and epidemiological surveys of human populations clearly showed the importance of Th2 responses for the control of gastrointestinal (GI) nematode infections^1–3^. However, type 2 responses develop slowly and inefficient protection against re-infection often results in lifelong chronic infections with GI nematodes in human populations, livestock and wild animals^4^. The small intestinal nematode *Heligmosomoides polygyrus* is a natural murine parasite that establishes chronic primary infections in several mouse lines and thus provides a suitable model for investigating long-lasting infections with GI nematodes in humans^5^. The infection results in the differentiation of Th2 cells releasing interleukin-4 and IL-13 along with strong B cells responses primarily characterized by IgG1 production. IL-4/-13 promote mucus production and the release of effector molecules by goblet cells as well as the alternative activation of macrophages. In concert, these responses eventually permit the expulsion of adult worms and provide protection against challenge infections^1,5^. Parasite clearance is sharply impaired in mice lacking CD4+ T cells or IL-4Rα^1,6,7^. Furthermore, inbred mouse lines such as C57BL/6 and BALB/c differ in resistance, i.e., the time required to control the infection and the quantity of eggs released by adult worms^7^. This was shown to correlate with the extent of both innate and adaptive type 2 responses, and likely also involves discrete differences in the activity of regulatory T cells^7,8^.

In accordance with the concept defining Th2 and Th1 generation as the result of opposing differentiation programs, high doses of type 1 cytokines applied during infections with intestinal nematodes were shown to block Th2 development and to severely impair the control of primary as well as challenge infections with GI nematodes^9,10^. Similarly, our group showed that Th2 differentiation in response to *H. polygyrus* infection is prevented in mice harboring high frequencies of IFN-γ competent Th1 and CD8+ T cells as a result of preceding exposure to *Toxoplasma gondii* infection^11^. Furthermore, we showed that, irrespective of the pre-exposure to other infections, a high proportion of naïve T cells activated in primary *H. polygyrus* infection differentiates into GATA-3+ T-bet+ Th2/1 hybrid cells^12^ and that IFN-γ signals integrated along with IL-4Rα signaling are critical for the commitment to the Th2/1 hybrid phenotype^13^. Th2/1 hybrid cells stably express T-bet along with intermediate GATA-3 expression and produce IFN-γ together with modest amounts of Th2 cytokines^12,13^. In line with the modest contribution of Th2/1 cells to IL-4 and IL-13 production and their identification as the main source of parasite-specific IFN-γ production, expanded Th2/1 hybrid cells lead to a further delay in the control of infection in highly susceptible C57BL/6 mice^12^.

Based on the importance of IFN-γ signaling for Th2/1 hybrid cell commitment and because IFN-γ competent memory-phenotype CD4+ and CD8+ T cells expand independent of pathogen exposure in an age-dependent manner^14,15^, the current study addressed if the phenotypical composition of GATA-3+ T effector cells generated in nematode-infected mice differed depending on host age. Investigating BALB/c mice displaying high genetic resistance to *H. polygyrus* infection, we show that IFN-γ competence of small intestinal CD4+ T cells increased with age and correlated positively with parasite fitness. Resistance was further increased after blocking of type 1 cytokines early during infection, whereas IFN-γ supplementation during priming of the CD4+ T cell response selectively promoted the outgrowth of Th2/1 hybrid cells, resulting in the impaired control of nematode fitness. The IFN-γ-Th2/1 hybrid-susceptibility axis was also evident in comparing partially resistant BALB/c to fully susceptible C57BL/6 mice, the latter exhibiting stronger accumulation of Th1 at steady state, more robust Th2/1 differentiation upon infection and a significant rise in resistance upon blocking of early type 1 cytokine signals.

## Results

### Age-dependent IFN-γ competence affects the phenotype of the type 2 response

**Fig. 1:**
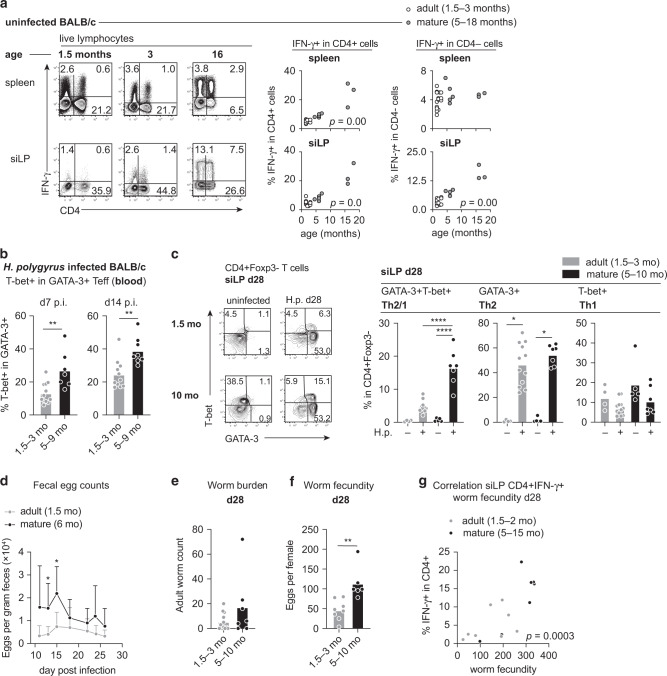
IFN-γ competence of BALB/c mice rises with age and reduces resistance to Th2-controlled *H. polygyrus* infection. **a** Representative contour plots depicting PMA/ionomycin-induced IFN-γ expression by CD4+ and CD4− cells derived from the spleen and small intestinal lamina propria (siLP) of naïve BALB/c mice at the age of 1.5, 3 and 16 months. Graphs depict correlation between the percentage of IFN-γ+ cells detected in CD4+ and CD4− cells and mouse age. Data from multiple independent experiments are pooled (*n* = 23–24 mice). **b** Frequencies of T-bet expressing cells in CD4+ GATA-3+ cells in blood at day 7 and day 14 post infection with *H. polygyrus*. Data from two independent experiments are pooled (*n* adult = 11–12; mature = 7–8 mice). **c** Representative flow cytometry plots depicting T-bet vs. GATA-3 expression in siLP-derived CD4+ FoxP3− cells at day 28 post *H. polygyrus* infection. Graphs report the frequencies of Th2/1, Th2 and Th1 cells in CD4+ FoxP3− siLP cells. Data from two independent experiments are reported (*n* adult naïve = 4; mature naïve = 5; infected adult = 12; infected mature = 7 mice). **d** Kinetics of fecal egg counts per gram feces over the course of infection in adult and mature mice. Data derive from one out of two experiments with similar results (*n* adult = 6; mature = 4 mice) and are shown as mean ± SD. **e** Adult worm counts at day 28 post infection. Data from two independent experiments are pooled (*n* adult = 12; mature = 7 mice). **f** Average number of eggs produced by individual female worms (typically eight worms per mouse, minimum 3) isolated from each mouse at day 28 post infection and cultured for 24 h. Data from two independent experiments are pooled (*n* adult = 8; mature = 6 mice; note that expulsion was completed in 4 adult and 2 mature mice). **g** Scatter graphs depict the correlation between the percentage of IFN-γ+ in siLP CD4+ T cells determined after PMA/ionomycin stimulation at day 28 post infection, and the mean egg counts produced by worms from the same mouse. Data derive from four experiments (*n* = 16). Bars in **b**, **c**, **e**, and **f** report the mean, circles represent individual mice. Statistical analysis was performed using Pearson correlation or nonparametric Spearman correlation analysis depending on the normality tests in a and g; unpaired *t* test or Mann–Whitney test in **b**, **d** and **f**; one-way ANOVA with Tukey’s multiple comparisons test or Kruskal–Wallis test with Dunn’s multiple comparisons test in **c**. *p ≤ 0.05, **p ≤ 0.01, ****p ≤ 0.0001.

In previous work, we identified IFN-γ as the main factor promoting the differentiation of Th2/1 hybrid cells in C57BL/6 mice infected with *H. polygyrus*^12,13^. Here, we asked whether the rise in IFN-γ competence resulting from the spontaneous generation and expansion of memory-phenotype (MP) Th1 cells and CD8+ T cells after birth^16^ might predispose the host for more extensive Th2/1 hybrid formation upon first encounter with an enteric nematode infection. BALB/c mice displaying high genetically controlled resistance to *H. polygyrus* infection were used to estimate the IFN-γ competence of lymphocytes isolated at steady state covering an age range of 1.5 up to 18 months according to PMA/ionomycin induced IFN-γ production (Fig. 1a). In spleen, rising IFN-γ signals derived primarily from CD4+ T cells and the percentage of CD4+ IFN-γ+ cells correlated positively with age (Fig. 1a). A similar rise in IFN-γ competence was observed in cells isolated from the small intestinal lamina propria (siLP), including a significant rise in IFN-γ+ CD4− cells (Fig. 1a). Furthermore, CD4+ CD44+ CD62-L− effector memory-like T cells isolated from spleen, mLN and siLP of naïve BALB/c mice at the age of 5.5 months were enriched in T-bet+ cells in comparison to cells from 3-month-old mice (Supplementary Fig. S1). Th2 cells surveyed side-by-side with Th1 cells in naïve mice did not change significantly over time (data not shown).

Next, we compared systemic and local T helper cell responses to infection with *H. polygyrus* in BALB/c mice infected at the age of 1.5–3 months or 5–9 months. GATA-3+ cells circulating in blood of mature mice comprised significantly higher proportions of T-bet co-expressing Th2/1 hybrid cells at day 7 and 14 post infection (Fig. 1b). At four weeks post infection, the early systemic shift in favor of Th2/1 cells observed in blood of the older cohort was reflected in higher frequencies of GATA-3+ T-bet+ cells populating the siLP of mature compared to young adult mice (Fig. 1c). More prominent Th2/1 cells in the small intestine of mature mice further coincided with elevated fecal egg counts shed by mature mice within the first week following the onset of egg deposition (Fig. 1d). Worm expulsion, typically occurring between week 4–6 post infection in BALB/c mice^5^, was unimpaired, as indicated by similarly low adult worm counts maintained in both age groups at day 28 post infection (Fig. 1e). However, female worms recovered from chronically infected mature mice produced significantly more eggs compared to females isolated from young adult mice (Fig. 1f). The effect of host age on parasite fitness was associated with a rise in local IFN-γ competence, indicated by the positive correlation between frequencies of IFN-γ+ small intestinal CD4+ T cells and female fecundity (Fig. 1g). Asking if the higher Th2/1 hybrid proportions detected in blood of mature mice early during infection developed at the expense of GATA-3 single-expressing Th2 cells, we compared the phenotype of T helper cells isolated from mesenteric lymph nodes (mLN) and spleen at day 6 post infection and found comparable frequencies of classical Th2 cells in both age groups (Supplementary Fig. S2) Despite the comparable instruction of Th2 cells in mLN, fewer Th2 cells homed to the small intestine in mature mice. In parallel, both mLN and spleen harbored higher frequencies of GATA-3+ T-bet+ Th2/1 cells as well as T-bet+ Th1 cells in mature mice (Supplementary Fig. S2). Taken together, these data show that Th2 differentiation is accompanied by the generation of more extensive Th2/1 hybrid responses in mature mice and that the accumulation of mucosal IFN-γ competent cells associates with the impaired control of parasite reproduction in mature compared to young adult mice.

### Early type 1 cytokine signals impact the control of nematode fitness in naturally resistant BALB/c mice

**Fig. 2:**
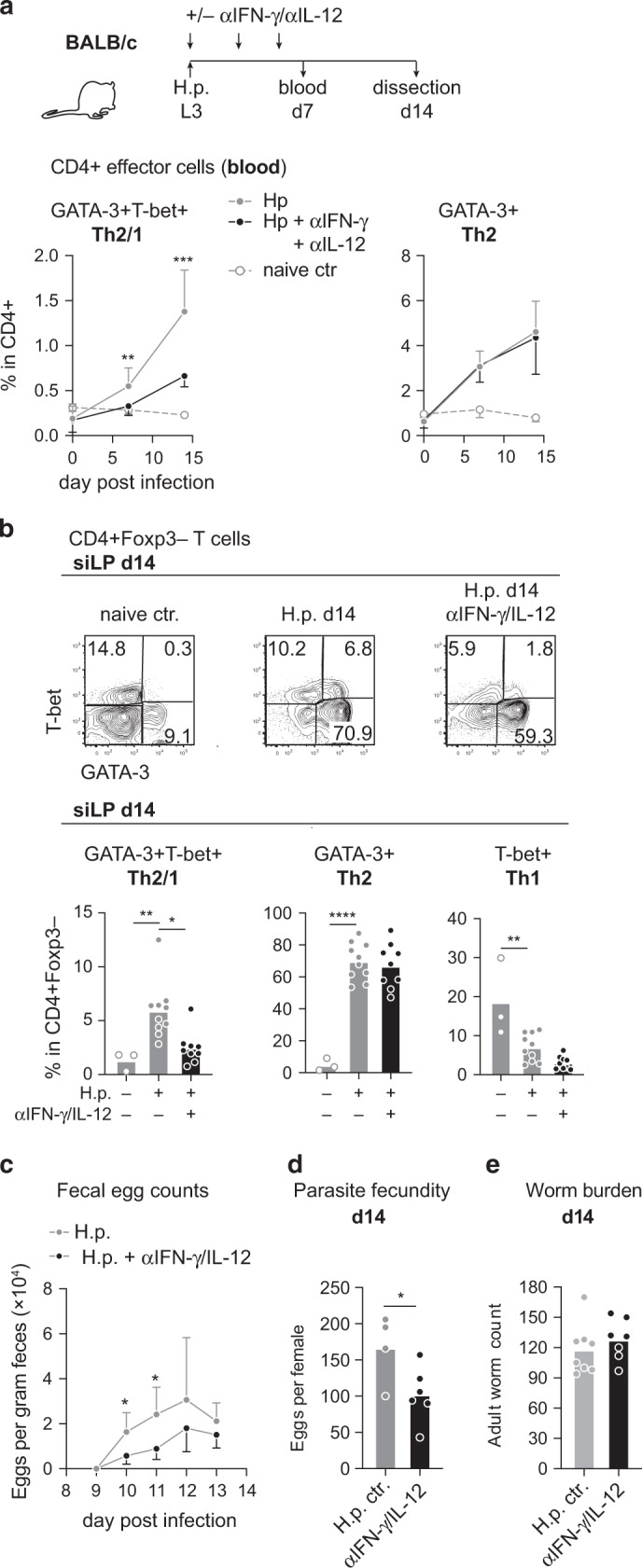
Blocking of early IFN-γ/IL-12 signals further promotes resistance to *H. polygyrus* infection in BALB/c mice. **a** Experimental setup: BALB/c mice were infected with *H. polygyrus* at the age of 1.5 months and treated with blocking antibodies against IL-12 and IFN-γ (0.5 mg each) at days 0, 3 and 6 post infection. Mice were dissected at day 14 post infection. Graphs report the frequencies of Th2/1 hybrid and Th2 cells (mean ± SD) determined in CD4+ cells in peripheral blood on day 0, 7 and 14. Data from three independent experiments are pooled. *n* naive = 2; Hp ctr = 11; αIFN-γ/αIL-12 = 10 mice. Samples with low PBMC counts were excluded. **b** Representative flow cytometry plots of T-bet and GATA-3 expression in siLP-derived CD4+ FoxP3− T cells isolated on day 14 post infection. Graphs depict the frequencies of Th2/1, Th2 and Th1 cells in CD4+ FoxP3− cells. Data from three independent experiments are pooled (*n* naïve = 3; Hp ctr = 10; αIFN-γ/αIL-12 = 9 mice). Samples with poor viability were excluded. **c** Fecal egg counts determined over the course of infection expressed as mean ± SD. Data derive from one out of three experiments with similar results (*n* Hp ctr = 4; αIFN-γ/αIL-12 = 6 mice). **d** Circles depict the mean egg production of 8 individual female worms per mouse within 24 h of culture. Data derive from one out of three independent experiments (*n* Hp ctr = 4; αIFN-γ/αIL-12 = 6 mice). **e** Adult worm burden at day 14 post infection. Data from two independent experiments are pooled (*n* Hp ctr = 8; αIFN-γ/αIL-12 = 7 mice). Bars in **b**, **d** and **e** report the mean, circles represent individual mice. Statistical analysis was performed using unpaired *t* test or Mann–Whitney test in **a**, **c**, and **d** and one-way ANOVA with Tukey’s multiple comparisons test or Kruskal–Wallis test with Dunn’s multiple comparisons test in **b**. *p ≤ 0.05, **p ≤ 0.01, ***p ≤ 0.001, ****p ≤ 0.0001.

To substantiate the importance of type-1 cytokines in the modulation of type-2 responses in *H. polygyrus* infection, we infected adult mice at the age of 1.5 months and applied blocking antibodies against IFN-γ and IL-12 on day 0, 3 and 6 of infection (Fig. 2a). The simultaneous interference with both IFN-γ and IL-12 signaling led to the selective and highly significant reduction of Th2/1 hybrid cells in blood as well as small intestine without affecting the accumulation of classical GATA-3+ Th2 cells or the numbers of Th1 cells in the infected gut (Fig. 2a, b). Worm counts determined at day 14 post infection were similar in both groups, but fewer circulating and mucosal Th2/1 cells coincided with significantly lower egg shedding and the reduced egg production by individual female worms isolated from the cytokine blocked group (Fig. 2c–e).

**Fig. 3:**
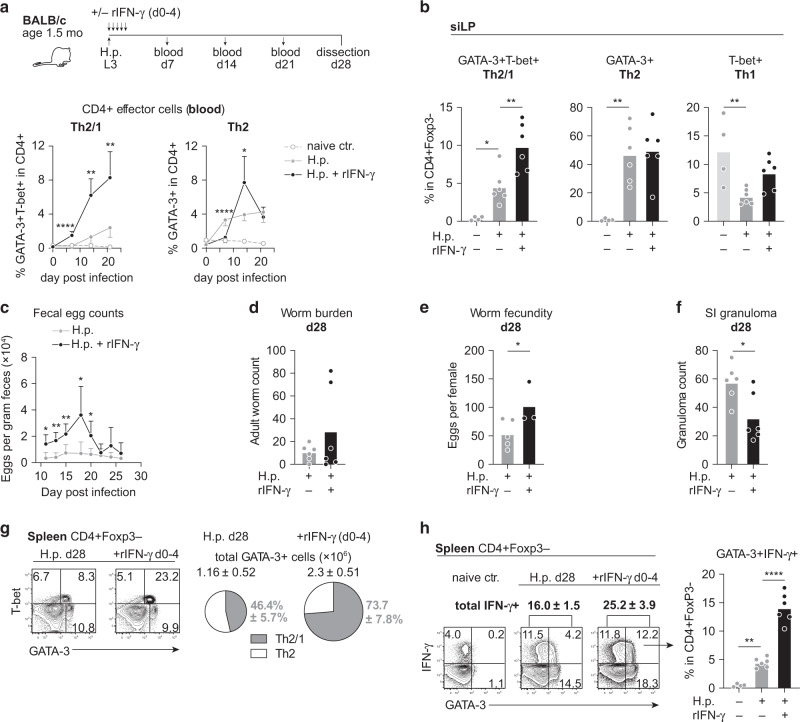
BALB/c mice supplemented with IFN-γ display impaired resistance associated with strongly expanded Th2/1 hybrid responses. **a** Experimental setup. BALB/c mice were infected with *H. polygyrus* at the age of 1.5 months and treated with recombinant IFN-γ (2.5 μg twice a day) from day 0 to 4 post infection. Mice were dissected at day 28 post infection. Graphs report the frequencies of Th2/1 hybrid and Th2 cells in CD4+ cells in blood over the course of infection. Data from two independent experiments are shown (*n* naïve = 3, infected ctr/ rIFN-γ treated = 6 mice) as mean ± SD. **b** Bar graphs depict the frequencies of Th2/1, Th2 and Th1 cells in siLP CD4+ T cells. Data from two independent experiments are pooled (*n* naïve = 4; infected ctr/ rIFN-γ treated = 6 mice). **c** Fecal egg counts determined over the course of infection. Data from two independent experiments are pooled (*n* = 6 mice/group) and shown as mean ± SD. **d** Adult worm counts at day 28 post infection. Data from two independent experiments are pooled (*n* = 6 mice/group). **e** Average number of eggs produced by individual female worms (typically eight worms per mouse, minimum 3) isolated from each mouse. Data from two independent experiments are combined (*n* infected ctr = 5; rIFN-γ treated = 3 mice). Note that expulsion was completed in one infected ctr and three rIFN-γ treated mice. **f** Granuloma count in small intestine at day 28 post infection. Data from two independent experiments are pooled (*n* = 6 mice/group). **g** Representative flow cytometry plots depicting T-bet and GATA-3 expression in CD4+ FoxP3− cells in spleen. Pie charts report the percentage of GATA-3+ T-bet+ Th2/1 cells in CD4+ GATA-3+ T cells isolated from the spleen at day 28. The size of the pie charts is adjusted according to the absolute cell count of GATA-3+ cells given above. Data from two independent experiments are pooled (*n* = 6 mice/group). **h** Representative flow cytometry plots depicting IFN-γ and GATA-3 expression of PMA/Ionomycin stimulated spleen cells at d28 post infection. Bar graph shows the frequencies of GATA-3+ IFN-γ+ cells in CD4+ FoxP3− cells isolated from spleen. Data from two independent experiments are pooled (*n* naïve = 4; infected ctr/rIFN-γ treated = 6 mice). Bars in **b**, **d**, **e**, **f** and **h** report the mean, circles represent individual mice. Statistical analysis was performed using unpaired *t* test or Mann–Whitney test in **a**, **c**, **e** and **f** and one-way ANOVA with Tukey’s multiple comparisons test in **b** and **h**. *p ≤ 0.05, **p ≤ 0.01, ****p ≤ 0.0001.

To see if the highly resistant phenotype of young adult BALB/c mice was altered by augmenting IFN-γ availability early during infection, we followed the reverse approach and treated BALB/c mice (1.5 months) with rIFN-γ twice daily (2.5 μg/dose) until day 4 post infection (Fig. 3a). IFN-γ supplementation resulted in a drastic rise of circulating GATA-3+ T-bet+ Th2/1 cells while a significant expansion of Th2 cells was only seen in blood once treatment had stopped (Fig. 3a). At four weeks post infection, the strong rise in IFN-γ-driven systemic Th2/1 responses was reflected in significantly more Th2/1 cells populating the small intestine (Fig. 3b). Classical Th2 cells accumulated to a similar extent in the intestines of treated as well untreated mice, indicating that the initial delay in Th2 expansion evident in blood was fully compensated for once treatment had stopped (Fig. 3b). In compliance with fostered mucosal Th2/1 hybrid accumulation, IFN-γ treated mice displayed elevated fecal egg counts during the first week of patency (Fig. 3c). Reflecting the pattern determined in mature versus young adult mice (Fig. 1), worm expulsion was not significantly impaired by the accumulation of more Th2/1 hybrid cells (Fig. 1d), but female worms retrieved from chronically infected mice that had initially been exposed to IFN-γ treatment displayed elevated fitness in higher egg deposition (Fig. 3e). In addition, significantly reduced numbers of IL-4/-13-dependent granuloma at the site of larval development indicated impaired local type 2 responses in IFN-γ treated mice (Fig. 3f). It is also noteworthy that IFN-γ supplementation during priming of the anti-nematode response led to a remarkable rise in frequencies as well as absolute counts of systemic GATA-3+ T cells (Fig. 3g). This was primarily due to the strong expansion of Th2/1 hybrid cells and was reflected in a drastic rise in IFN-γ production by GATA-3+ cells isolated from the spleen at day 28 post infection (Fig. 3h). Together, these data show that blocking early type 1 cytokine signals further augments the high resistance of BALB/c mice to *H. polygyrus* infection. Conversely, elevated IFN-γ availability early during infection results in impaired control of parasite fitness, which is associated with the sustained systemic expansion and mucosal accumulation of IFN-γ competent Th2/1 cells.

### The phenotype of parasite-specific effector T cells is highly sensitive to differential IFN-γ availability

**Fig. 4:**
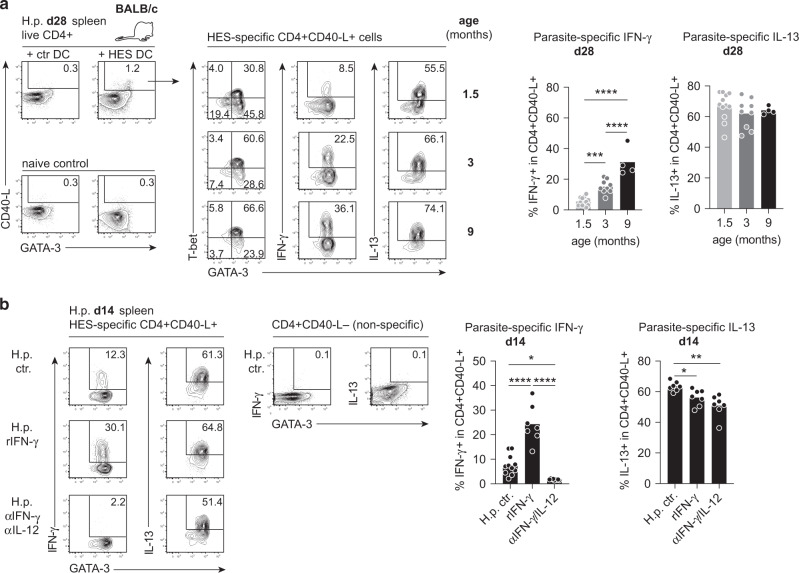
IFN-γ production by parasite-specific CD4+ T cells differs depending on host age and early IFN-γ availability. The phenotype of parasite-specific CD4+ T cells was determined after stimulation of spleen cells with *H. polygyrus* excretory/secretory products (HES) loaded on bone-marrow derived dendritic cells (DC). **a** Selective upregulation of CD40-L expression by CD4+ T cells derived from chronically infected BALB/c mice in cultures with HES-loaded DC (left side). CD4+ T cells of naïve control mice (lower row) did not respond to HES-DC. Plots on the right depict GATA-3 and T-bet expression (first column), IFN-γ expression (center) and IL-13 expression (right column) in CD40-L+ cells. CD40-L negative cells did not comprise cytokine expressing cells (not shown). All plots were generated by merging the data of three mice per age group. Graphs report the frequencies of IFN-γ+ and IL-13+ cells determined in CD40-L+ cells of 1.5 mo (*n* = 12), 3 mo (*n* = 9) and 9 mo (*n* = 4) old mice as determined in four independent experiments. **b** Plots report the percentages of IFN-γ (1^st^ column) and IL-13 producing cells (2^nd^ column) determined in CD40-L+ cells (left) and CD40-L− cells (right). Graphs reports the frequencies of IFN-γ+ and IL-13+ cells determined in CD40-L+ cells of untreated infection controls (*n* = 7–11), rIFN-γ supplemented (*n* = 7) and αIL-12/αIFN-γ treated mice (*n* = 7) as determined in two to three independent experiments. Bars report the mean, circles represent individual mice. Statistical analysis was performed using one-way ANOVA with Tukey’s multiple comparisons test. *p ≤ 0.05, **p ≤ 0.01, ***p ≤ 0.001, ****p ≤ 0.0001.

To corroborate the relevance of the distinct responses determined depending on host age, we assessed the phenotype of parasite-specific CD4+ T cells isolated from mice infected with *H. polygyrus* at the age of 1.5, 3 or 9 months (Fig. 4a). Splenocytes isolated at day 28 post infection were cultured with dendritic cells (DC) presenting excretory/secretory products of *H. polygyrus* (HES). Parasite-specific CD4+ T cells responding to TCR activation by the upregulation of CD40-ligand (CD40-L) were surveyed for the expression of GATA-3/IL-13 and T-bet/IFN-γ. The vast majority of parasite-specific CD40-L+ cells expressed GATA-3 in all age groups (Fig. 4a). CD40-L+ cells retrieved from the youngest group were dominated by GATA-3+ Th2 cells and a few produced IFN-γ (Fig. 4a). In strong contrast, HES-responsive cells of mice infected at the age of 3 or 9 months comprised significantly more GATA-3+ T-bet+ Th2/1 cells and, consequently, far more cells producing high levels of IFN-γ (Fig. 4a). Furthermore, young adult mice that had been transiently exposed to IFN-γ during primary encounter with GI nematodes displayed a highly significant rise of IFN-γ producing parasite-specific cells, whereas experimental blocking of early type 1 cytokines almost completely prevented the generation of IFN-γ+ HES-responsive cells (Fig. 4b). While the mean percentage of IL-13+ cells within the CD40-L+ population did not differ depending on host age, IFN-γ supplementation or blocking of type 1 signals led to a modest, but significant decline in IL-13 production by parasite-specific cells (Fig. 4a, b). Collectively, these data suggest that the rise in IFN-γ competent cells associated with immune maturation, but also transient episodes of high IFN-γ availability (e.g., in a coinfection setting) may profoundly alter the outcome of type 2 effector cell differentiation in the nematode-infected host, which in turn impacts the control of parasite reproductive fitness.

### Increased local IFN-γ competence after IFN-γ supplementation affects larval fitness

**Fig. 5:**
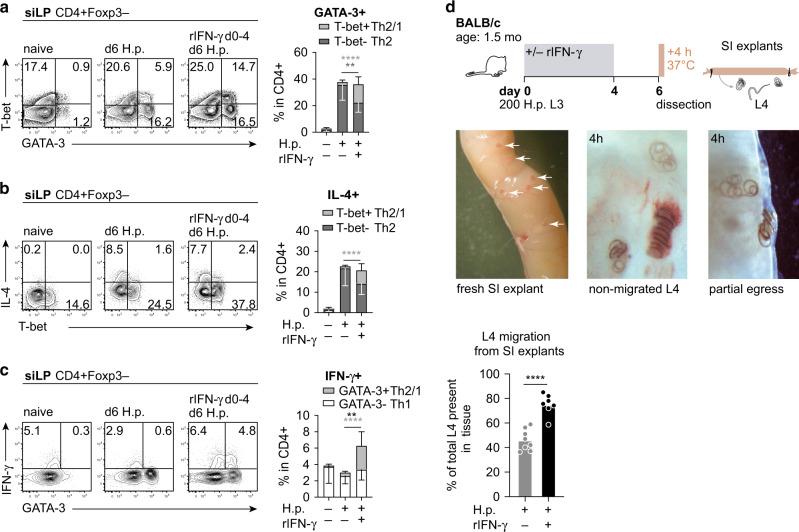
IFN-γ supplementation promotes early intestinal accumulation of Th2/1 cells and impacts larval fitness. Adult BALB/c mice (1.5 mo) were infected with *H. polygyrus* and treated with rIFN-γ (2.5 μg twice a day) during the first five days of infection and dissected at day 6 post infection. T-bet and GATA-3 expression (**a**), IL-4 and T-bet expression (**b**) and IFN-γ versus GATA-3 expression (**c**) in siLP-derived CD4+ FoxP3− cells. Stacked bars report the percentage of total GATA-3+, IL-4+ and IFN-γ+ cells, deciphering between T-bet+/− and GATA-3+/− cells as indicated. Cells were stimulated with PMA/ionomycin. Data from three independent experiments (*n* naïve = 6; *n* infected = 9–10 mice/group) are reported as mean ± SD. **d** Experimental setup. Small intestinal explants were cultured at 37 °C and the number of 4^th^ stage larvae migrating from host tissue was determined after 4 h. Pictures show a freshly prepared small intestinal explant with L4 embedded in the tissue (white arrows); L4 remaining in the tissue after 4 h of cultivation; and partially egressed 4^th^ stage larvae. Graph reports L4 migrated from the tissue within 4 h (mean and individual mice), expressed as the percentage of total larvae in the tissue explant. Data from two independent experiments are pooled (*n* = 7–8 mice/group). Bars report the mean, circles represent individual mice. Statistical analysis was performed using one-way ANOVA and Tukey’s multiple comparisons test or Kruskal–Wallis test and Dunn’s multiple comparisons test in **a**–**c** and unpaired *t* test in **d**. The statistical significance comparing each sub-population in **a**–**c** is shown by asterisks in the corresponding colors. Black asterisks in **c** indicate significantly different overall frequencies of IFN-γ+ producing cells comparing infection controls and IFN-γ-treated mice. **p ≤ 0.01, ****p ≤ 0.0001.

Worm fitness, estimated according to egg release by adult females, correlated with IFN-γ production by intestinal T cells and the accumulation of IFN-γ competent Th2/1 cells at the site of infection (Figs. 1–3). Hence, we hypothesized that the inflammatory environment experienced by tissue-standing larvae might translate to differences in reproductive fitness after transition to the adult stage (Figs. 1f, 2d and 3e). Investigating the early intestinal T cell responses of IFN-γ-treated mice and untreated controls in further detail, we found that early exposure to IFN-γ led to decline in the frequencies of classical Th2 cells in siLP at day 6 post infection and a compensatory rise in early Th2/1 hybrid cell accumulation (Fig. 5a). Accordingly, IL-4 signals were similar between the two groups, whereas the higher proportions of Th2/1 cells translated to significantly more robust IFN-γ production upon restimulation (Fig. 5b, c). To see if these changes were associated with altered larval fitness, we adopted a method used for the isolation of intact fourth stage larvae (L4) from intestinal tissue^17^. Taking advantage of the inverted migratory behavior of L4 to the abluminal side of ligated small intestinal tissue explants, we found that a significantly higher percentage of L4 managed to fully exit the cultured explants of IFN-γ-treated mice compared to those of untreated infection controls within four hours (Fig. 5d). Hence, the accumulation of intestinal IFN-γ competent Th2/1 hybrid cells appears to facilitate the egress of nematode larvae from host tissue, suggesting an advantage in larval fitness which translates to increased egg production by adult worms shortly later.

### Pronounced Th1 accumulation at steady state and strong Th2/1 hybrid responses in susceptible C57BL/6 mice

**Fig. 6:**
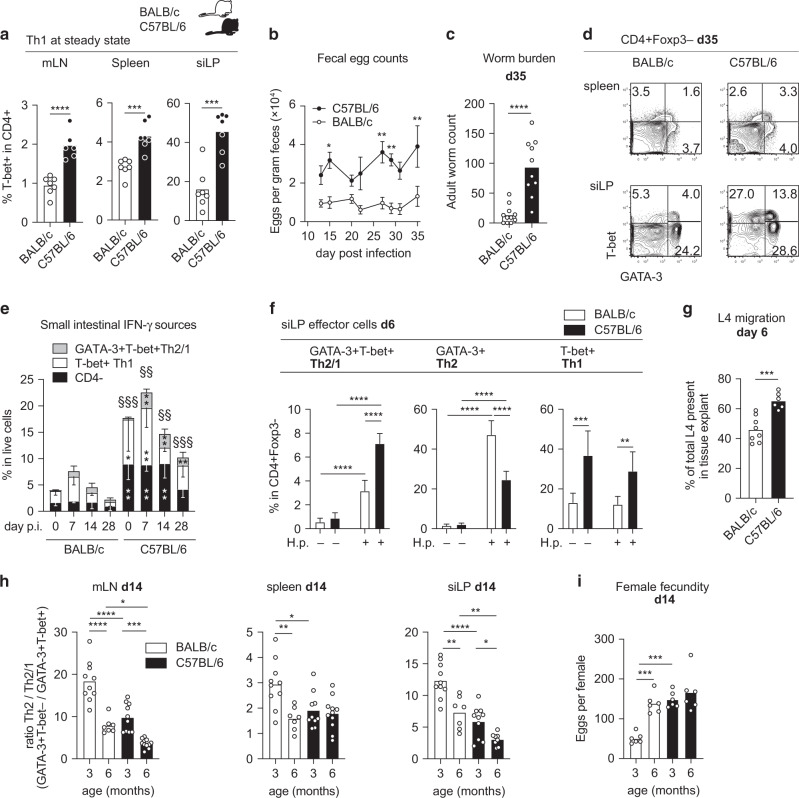
Highly susceptible C57BL/6 mice display more extensive local Th2/1 hybrid accumulation next to more prominent Th1 cells. **a** Frequencies of T-bet expressing cells in CD4+ cells in mLN, spleen and small intestine of naïve BALB/c and C57BL/6 mice at the age of 1.5 months. Data from three independent experiments (*n* = 6–7 mice/group) are pooled. **b** Fecal egg counts of *H. polygyrus* infected BALB/c and C57BL/6 mice over the course of five weeks. Data derive from one out of two experiments with similar outcome (*n* = 5 mice/line) and are reported as mean ± SD. **c** Adult worm counts at day 35 post infection. Data from three independent experiments (*n* = 10–11 mice/group) are pooled. **d** T-bet and GATA-3 expression in CD4+ Foxp3− cells in spleen and small intestine at d35 post infection. Statistics are provided in Supplementary Fig. S3. **e** Stacked bars depicting small intestinal IFN-γ responses by CD4−, Th1 and Th2/1 cells shown from one dataset representative for 2-3 experiments as mean ± SD. *n* = 3–6 per infection time point. Naïve controls (*n* = 5 and 8) are pooled from four individual experiments. Significant differences between the mouse lines at a given time point concerning the total and cell type-specific IFN-γ signals are indicated by (§) and (*), respectively. **f** Frequencies of Th2/1, Th2 and Th1 cells in CD4+ FoxP3− cells in small intestine. Data from two (C57BL/6) or three (BALB/c) experiments are pooled (*n* BALB/c naïve = 7, C57BL/6 naïve = 4; BALB/c infected = 10, C57BL/6 infected = 6 mice) and reported as mean + SD. **g** Quantification of L4 migrated from small intestinal tissue explants within 4 h, expressed as percentage of the total larvae present in tissue. Data from two experiments each performed with four BALB/c and three C57BL/6 mice per group are pooled. **h** Ratio of Th2 to Th2/1 cells at day 14 in mLN, spleen and small intestine. Data from 2 to 3 individual experiments each performed with 3–5 mice/group are reported. **i** Mean egg production of individual female worms within 24 h of culture. Data from 2 independent experiments each performed with *n* = 3 mice/group are pooled. Bars in **a**, **c**, **g**, **h** and **i** report the mean, circles represent individual mice. Statistical analysis was performed using unpaired *t* test or Mann–Whitney test in **a**–**c**, **e** and **g**, and one-way ANOVA with Tukey’s multiple comparisons test or Kruskal–Wallis test with Dunn’s multiple comparisons test in **f**, **h** and **i**. *p ≤ 0.05, **p ≤ 0.01, ***p ≤ 0.001, ****p ≤ 0.0001.

Next, we asked if the higher susceptibility of ‘Th1-prone’ C57BL/6 compared to ‘Th2-prone’ BALB/c mice to *H. polygyrus* infection^5,7^ was associated with the more rapid accumulation of IFN-γ competent cells at steady state and more pronounced Th2/1 hybrid responses in the C57BL/6 line. Indeed, uninfected C57BL/6 mice (mean age: 1.9+/− 0.5 months) harbored significantly higher percentages of T-bet+ Th1 cells in spleen, mLN and siLP compared to naïve BALB/c mice with a similar age range (mean age: 2+/− 0.6 months) (Fig. 6a). As expected, infected C57BL/6 mice released more parasite eggs and maintained higher worm counts at 35 days post infection (Fig. 6b, c). This was associated with more robust Th2/1 hybrid responses in spleen and siLP at day 35, while the frequencies of Th2 cells were similar in both lines (Fig. 6d, Supplementary Fig. S3). Poor resistance of the C57BL76 line was further associated with higher frequencies of IFN-γ competent Th2/1, Th1 and CD4- cells in siLP along the course of infection (Fig. 6e). At day 6 post infection, the bias in favor of Th1 and Th2/1 cells in siLP of C57BL/6 compared to BALB/c mice translated to the more effective migration of fourth stage larvae from tissue explants of C57BL/6 mice (Fig. 6f, g). The early bias for Th2/1 cells in C57BL/6 mice was also evident when comparing the IFN-γ and T cell responses to highly resistant SJL strain (Supplementary Fig. S4). SJL mice, similar to BALB/c mice, displayed limited IFN-γ availability in mLN, spleen and siLP (Supplementary Fig. S4a), poor small intestinal Th2/1 accumulation at early stage of infection (Supplementary Fig. S4b) and stronger M2 polarization in the peritoneal cavity compared to susceptible C57BL/6 mice (Supplementary Fig. S4c).

Next, we compared classical Th2 and Th2/1 hybrid responses in both mouse lines infected at the age of three or six months and determined the ratio of Th2 to Th2/1 cells in lymphatic organs and small intestines at day 14 post infection (Fig. 6h). In both mouse lines, the mature groups responded with more prominent Th2/1 differentiation, evident in the significant drop of Th2 to Th2/1 hybrid cell ratios in mLN and siLP (Fig. 6h). Importantly, equal Th2 to hybrid ratios determined in mature BALB/c and younger C57BL/6 mice corresponded with similar worm fecundity at day 14, underlining the reliability of the fecundity readout across genotypes and age groups. The higher egg release by worms isolated from younger C57BL/6 compared to age matched BALB/c mice was further enhanced for worms isolated from the mature C57BL/6 group (Fig. 6i). Together, these data strongly suggest that genetically determined as well as age-related differences in IFN-γ availability impacts the phenotype of GATA-3+ T cells in mice infected with intestinal nematodes and that the pronounced differentiation of Th2/1 hybrid cells is associated with impaired resistance.

### The susceptible phenotype of C57BL/6 mice depends on early IFN-γ availability

**Fig. 7:**
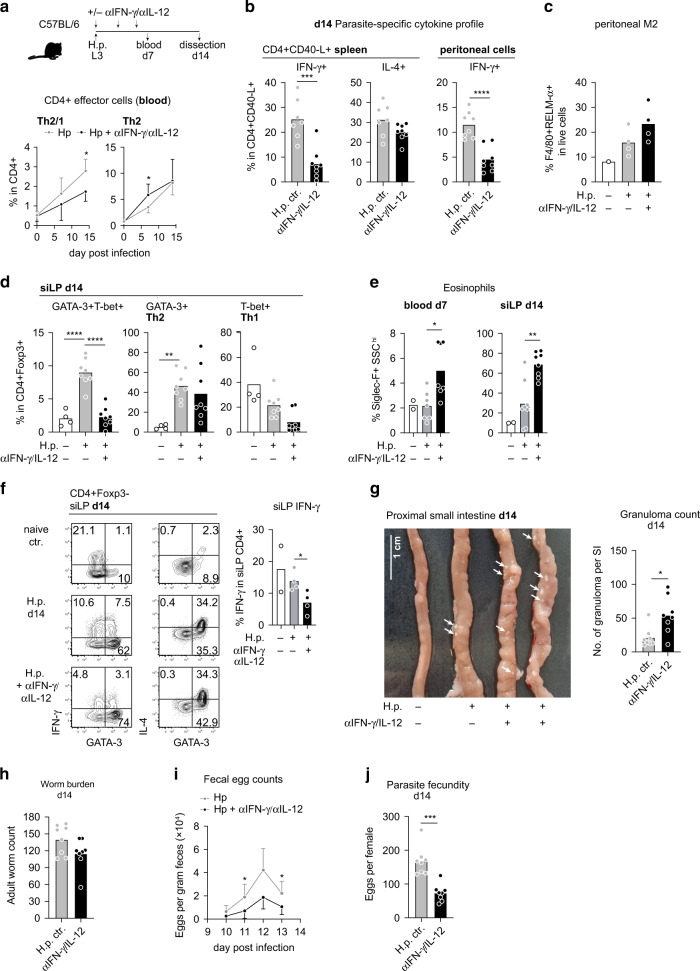
Blocking of early type 1 cytokine responses supports resistance of the susceptible C57BL/6 line. **a** Experimental setup: C57BL/6 mice (3.5 ± 2 months) were infected with *H. polygyrus* and treated with αIL-12 and αIFN-γ as described in figure legends. Experiments were terminated at day 14 post infection. Graphs depict the percentage of Th2/1 hybrid and Th2 cells in circulating CD4+ cells over the course of infection. Data from two independent experiments are pooled (*n* naïve = 6, *n* infected = 4–7 mice/group) and shown as mean ± SD. **b** Frequencies of IFN-γ and IL-4 producing cells in parasite-specific CD4+ cells in spleen and peritoneal cavity. Data from two independent experiments are pooled (*n* Hp ctr = 7–8; αIFN-γ/αIL-12 = 8 mice). **c** Percentage of F4/80+RELM-α+M2 macrophages in live peritoneal cells. Data derive from one experiment (*n* naïve = 1; Hp ctr = 4; αIFN-γ/αIL-12 = 4 mice). **d** Frequencies of Th2/1, Th2 and Th1 cells in CD4+ FoxP3- cells derived from small intestine. Data from two independent experiments are pooled (*n* naïve = 4; Hp ctr = 8; αIFN-γ/αIL-12 = 8 mice). **e** Bar graphs depict the frequencies of Siglec-F+SSC^hi^ eosinophils in blood and small intestine at indicated time points. Data from two independent experiments are pooled (*n* naïve = 2; Hp ctr = 7-8; αIFN-γ/αIL-12 = 8 mice). **f** Left: Plots depicting the IFN-γ and IL-4 expression in CD4+ FoxP3- cells in response to PMA/Ionomycin stimulation; Right: Bar graphs report the IFN-γ producing cells in small intestinal CD4+ cells. Data derive from one out of two experiments with similar outcome (*n* naïve = 2; Hp ctr = 4; αIFN-γ/αIL-12 = 4 mice)**. g** Representative pictures of the proximal small intestines of one naïve and one *H. polygyrus* infected control mouse compared to the SI of two mice that had received blocking antibodies to IFN-γ and IL-12 early during the infection. Arrowheads point at granuloma. The combined data of two experiments are reported in the bar graph (*n* Hp ctr = 8; αIFN-γ/αIL-12 = 8 mice). **h** Adult worm burden at day 14 post infection. Data from two independent experiments are pooled (*n* Hp ctr = 8; αIFN-γ/αIL-12 = 8 mice). **i** Fecal egg counts determined from day 10 to 13 post infection shown as mean ± SD. Data from two independent experiments are pooled (*n* = 8 mice per group) at each time point except day 12 where one experiment with *n* = 4 mice/group is shown. **j** Data points depict the mean egg production of 8 individual female worms per mouse within 24 h of culture. Data from two independent experiments are pooled. (*n* Hp ctr = 8; αIFN-γ/αIL-12 = 8 mice). Bars in **b**–**h** and **j** report the mean, circles represent individual mice. Statistical analysis was performed using unpaired *t* test or Mann–Whitney test in **a**, **b**, **e**–**g**, **i** and **j**, and one-way ANOVA with Tukey’s multiple comparisons test or Kruskal–Wallis test and Dunn’s multiple comparisons test in **d**. *p ≤ 0.05, **p ≤ 0.01, ***p ≤ 0.001, ****p ≤ 0.0001.

Finally, we asked whether blocking of type 1 cytokines during the onset of *H. polygyrus* infection was sufficient for converting the immunological phenotype of C57BL/6 mice to the more effective response seen in BALB/c mice. To that end, C57BL/6 mice were infected at the age of three months, treated with blocking antibodies against IFN-γ and IL-12 as described above and dissected at day 14 post infection. As seen in BALB/c mice (Fig. 2), the restricted availability of type 1 cytokines early during infection resulted in diminished proportions of Th2/1 cells in blood (Fig. 7a) and in significantly reduced frequencies of IFN-γ producing cells within the parasite-specific CD4+ CD40-L+ populations in spleen and peritoneal cavity (Fig. 7b). The reduction of systemic and peritoneal Th2/1 cells was associated with a modest, non-significant rise in RELM-α+ type 2 macrophages in the peritoneal cavity (Fig. 7c). More strikingly, limited systemic and intestinal Th2/1 hybrid responses (Fig. 7a, d) were accompanied by the more rapid rise in circulating eosinophils and the more pronounced accumulation of eosinophils in siLP at day 14 post infection (Fig. 7e). IL-4 production by intestinal CD4+ T cells was similar between the infected groups, but Th1- as well as Th2/1-derived IFN-γ production was diminished in treated mice (Fig. 7f). Finally, the low IFN-γ competence along with robust local IL-4 production licensed the formation of significantly stronger granuloma responses at the site of larval development (Fig. 7g) and allowed for the more effective control of parasite reproduction by Th2/1-deprived C57BL/6 mice (Fig. 7h–j). In summary, these data show that the high susceptibility of C57BL/6 mice to *H. polygyrus* infection is linked with the early accumulation of IFN-γ competent cells at steady state, ensuing in the generation of robust Th2/1 responses upon nematode infection. Importantly, the interference with early type 1 cytokine signaling and, consequently, the generation of extensive Th2/1 hybrid cell responses, not only further promotes resistance of the Th2-prone BALB/c line, but also permits for the more efficient control of parasite reproduction by genetically susceptible C57BL/6 mice.

## Discussion

The current study addressed whether a rise of IFN-γ competent Th1 cells along the maturation of the murine immune system impacts the development of effective type 2 responses in enteric nematode infections. We show that the age-dependent rise of IFN-γ competent cells seen in lymphatic organs as well as intestinal tissue at steady state correlates with more extensive Th2/1 hybrid differentiation in nematode-infected mature compared to young adult mice. Furthermore, IFN-γ treatment of young adult mice early during infection mirrors the effect of more advanced age in the promotion of Th2/1 cell differentiation and an associated rise in nematode fitness. As Th2/1 commitment critically depends on IFN-γ signals^13^, it is likely that the early release of IFN-γ is key to the observed age-dependent shifts within the nematode-induced GATA-3+ T cell pool. This is further corroborated by the finding of earlier accumulation of Th1 cells at steady state in the more susceptible C57BL/6 line. These mice generate stronger Th2/1 hybrid response when infected at a young adult age compared to age matched BALB/c mice, which translates to higher parasite egg release. Importantly, the blocking of early type-1 cytokine signals restrains Th2/1 hybrid generation in both mouse lines and allows for the more efficient control of parasite reproduction by the genetically resistant as well as the more susceptible host.

Focusing on BALB/c mice displaying a genetic bias for the induction of robust Th2 responses to nematode as well as *Leishmania* infections^7,18–20^, we confirm that IFN-γ competent Th1 cells expand significantly with age in lymphatic organs and intestinal tissue at steady state, an observation previously reported for C57BL/6 mice^16,21^. Such memory phenotype (MP) Th1 cells expand after birth, reaching a plateau at the age of six months. MP Th1 induction is not contingent to the presence of foreign antigens, but depends on IL-12 constitutively produced by CD8α+ dendritic cells^16,21^. MP Th1 were reported to rapidly produce IFN-γ in an innate-like manner in the context of *Toxoplasma gondii* infection, and thereby support the development of adaptive Th1 responses^16^. Hence, while MP Th1 cells were shown to be beneficial in the context of *Toxoplasma* infection, our data suggest that the expansion of MP Th1 cells along age exerts detrimental effects on the development of anti-nematode Th2 responses.

Importantly, several studies reported the expression of type 1 factors that may support IFN-γ release by MP Th1 as well as CD8+ and NK cells in the context of helminth infections. This comprises the constitutive expression of IL-12 by CD103+ migratory dendritic cells and the release of IL-1β and IL-18 by macrophages responding to nematode products or helminth driven tissue damage^22–25^. IL-12 and IL-18 synergize in driving IFN-γ production by Th1 and NK cells^26^ and the inflammasome dependent release of IL-18 was previously shown to promote IFN-γ production by CD4+ T cells in whipworm infected mice^24^. Furthermore, several recent studies reported a type-1 signature, including the production of IFN-γ, in small intestinal tissue surrounding the larval stage of *H. polygyrus*^27–29^. It hence seems likely that higher numbers of Th1 cells present in mature mice drive the more vigorous Th2/1 hybrid responses in nematode-infected mature compared to younger individuals, resulting in the less efficient control of parasite reproduction. Compliant with the differential resistance of the two mouse lines investigated here, we further show that the accumulation of Th1 MP-like cells is delayed in BALB/c compared to C57BL/6 mice. This may relate to the poor maintenance of the IL-12Rβ2 chain and the more limited IL-12 production by antigen presenting cells reported for the BALB/c compared to other mouse lines^30,31^.

Previous studies showed that type 2 responses are impaired in mice simultaneously infected with helminths and Th1-inducing pathogens^11,32–34^. Similarly, high doses of IFN-γ or IL-12 applied early during nematode infection result in poor Th2 differentiation and delayed worm expulsion^6,9,35^. Furthermore, the IL-4 driven inability of BALB/c mice to control *Leishmania* infections was shown to be confined to young animals, whereas old mice controlled the infection, partially depending on elevated IL-12 production by macrophages in aged mice^36^. Similarly, defective Th2 differentiation and strongly Th1-biased responses were reported for nematode-infected senescent mice (age ≥ 1.5 years)^37–40^. Our study expands on these findings, suggesting that the age-dependent IFN-γ availability impacts the phenotype of the developing T cell response in nematode-infected mice and thereby impedes the type-2 dependent control of nematode fitness.

We further show that the divergent patterns seen in young adult BALB/c compared to C57BL/6 mice upon nematode infection tend to level out with progressing age of the BALB/c line, associated with the rise in Th1 cells at steady state. Of note, our demonstration of age- and IFN-γ dependent shifts of the immune response and resistance to a GI nematode infection is based on mice exposed to a single high dose infection under highly controlled environmental conditions. This does not necessarily imply that age-related differences have a similarly clear effect on the anti-helminth responses in natural systems. Our data may rather suggest that differential IFN-γ availability related to host age and genotype may take part in the modification of anti-helminth immune responses in e.g., wildlife and human populations. However, given that GI nematodes are typically acquired at a young age, IFN-γ availability depending on the coinfection status as well as the structure of the host microbiome may be more important and result in similar consequences for anti-helminth immunity in natural systems, which is as addressed further below.

Worm expulsion was largely intact in mature BALB/c mice exhibiting prominent mucosal Th2/1 hybrid accumulation and strong parasite-specific IFN-γ production. Nevertheless, egg release by adult worms proved a reliable correlate with the extent of intestinal Th2/1 hybrid accumulation depending on host age (Figs. 1 and 6), genotype (Fig. 6) and the manipulation of early IFN-γ levels (Figs. 2, 3 and 7). In addition, fecal egg counts typically differed from the onset of egg deposition by adult worms depending on IFN-γ availability. To see whether the composition of local effector T cells affected larval fitness, we adopted a protocol used to isolate 4^th^ stage larvae from small intestinal tissue^17^. Under the given culture conditions, larvae migrate towards the abluminal side^17^, whereas in situ migration back to the small intestinal lumen is accomplished around day eight post infection^5^. Although not reflecting the natural behavior, our finding of accelerated egress of the 4^th^ larval stage from host tissue infiltrated by more Th2/1 cells indicates that larval fitness is affected by the early local T cell responses, which translates to the differential reproductive fitness of the adult stage shortly later.

Notably, neither the elevated endogenous IFN-γ availability seen in mature mice, nor IFN-γ applied to young adult mice resulted in persistent defects in classical Th2 cell differentiation. Rather, BALB/c mice treated with IFN-γ early during infection displayed a remarkable rise of GATA-3+ effector cells due to much stronger Th2/1 hybrid responses (Fig. 3). Hence, while an earlier study showed that small amounts of IFN-γ are required for optimal in vivo priming of IL-4+ T cells and the antigen-driven expansion of IL-4+ cells in vivo^41^, the age-dependent rise in IFN-γ availability primarily promoted the number of IFN-γ competent Th2/1 cells within the parasite-specific T cell pool in our model (Fig. 4) and IFN-γ applied to adult nematode-infected mice resulted in the strong systemic Th2/1 hybrid responses, further reflected in the much stronger IFN-γ production by parasite-specific GATA-3+ cells (Figs. 3 and 4). Conversely, blocking of early IFN-γ signals led to a decline in Th2/1 cells and the nearly complete absence of GATA-3+IFN-γ+ parasite-specific cells next to significant, but rather modest decline in IL-13 production by parasite-specific T cells. Hence our data confirm the compatibility of IFN-γ signaling with Th2 differentiation^41^, but further show that IFN-γ signals integrated during T cell priming in a nematode infection primarily promote the expansion of GATA-3+ T-bet+ hybrid cells which translates to the impaired control of parasite fitness.

Our data further suggest an inverse relationship between IFN-γ availability and the IL-4Rα-dependent granuloma formation at the site of larval development^42^. This complies with a recent study showing that conditional ablation of IFN-γ responsiveness in intestinal glial cells leads to delayed resolution of the inflammatory intestinal granuloma in *H. polygyrus* infected mice^29^. Furthermore, the accumulation of IFN-γ+ lymphocytes and the associated type 1 transcriptional signature around the tissue-dwelling larvae was linked to remodeling of the intestinal stem cell niche and the recruitment of NK cells, allowing for the rapid repair of epithelial damage and preventing vascular injury, respectively^27,28^. Hence, IFN-γ signaling, either direct or conveyed by enteric neural cells, appears to be crucial for resolving nematode-induced tissue damage. The co-localization of IFN-γ competent Th2/1 hybrid cells with the large numbers of Th2 effector cells invading the nematode-infected gut may thus secure tissue integrity via IFN-γ production in situations where resident Th1 cells are outnumbered by Th2 effector cells.

Resistance to GI nematodes typically develops slowly along the rise of Th2 and antibody responses, but remains incomplete in human populations^4^, livestock^43^ as well as wild mammals^44^. Hence, the question remains whether differential IFN-γ availability depending on host age, the coinfection status with Th1-inducing pathogens and the host genetic background similarly affect the phenotype of the type-2 response and the control of GI nematodes in natural systems. Similar to murine MP Th1 cells, antigen-inexperienced CD4+ and CD8+ T cells releasing substantial amounts of IFN-γ upon cytokine or polyclonal triggers have been identified in humans and were shown to expand with age^15,45,46^. Furthermore, conventional Th1 and CD8+ memory T cells induced in humans and wild animals in response to viral, microbial and protozoan infections next to NK may serve as IFN-γ sources in response to cytokine released in the context of nematode infections. However, whether the differential release of type-1 cytokines along age or infection history affects the development of type-2 mediated resistance in natural systems is unknown. Most epidemiological surveys of human populations in helminth endemic areas show that resistance to GI nematodes rises with host age^3,47^, whereas several studies focusing on hookworm infections reported a rise in prevalence and infection intensities with age, with elderly patients displaying the highest egg counts and worm burdens^48–50^. Unfortunately, the cytokine profiles expressed by the different age groups were not determined. In contrast to hookworms, the infection intensities with *Ascaris* spp. and whipworms typically peak in school-age children and decline along the rise of Th2 responses before adulthood^51^. However, rising parasite-specific IFN-γ production along the age of 4–15 years reported for children infected with the whipworm *T. trichiura* may indicate the contribution of type-1 activity to the incomplete control of whipworm infections thereafter^52^. Similarly, while infection intensities with *Ascaris lumbricoides* decline along the development of Th2-biased parasite-specific responses^53^, the elevated expression of IL-12, STAT4 and IL-18 in ileum and liver as well as the elevated IFN-γ expression in lung tissue reported for *Ascaris*-infected pigs indicates an environment suited for the induction of IFN-γ expression in T and NK cells^54,55^. Furthermore, work in our group previously showed that the parasite-specific T cell pool generated in *Ascaris*-infected pigs comprises IFN-γ+ cells^56^. Hence, further work addressing the role of Th2/1 hybrid cells in the context of natural GI nematode infections is needed and the profiles of IL-4/IFN-γ expression by porcine *Ascaris*-specific CD4+ T cells are currently under investigation in our group.

Finally, the immune system of mice kept under specific pathogen-free conditions differs dramatically from mice maintained in a barrier free environment, as recently reviewed by Hamilton et al.^57^, and captured wild house mice exhibit increased proportions of effector/memory T cells that release IFN-γ upon re-stimulation and their NK cells display a more active state^58^. This may indicate that the high prevalence of GI nematodes detected in populations of wild rodents relates to strong Th1/NK cell activity impeding the generation of efficient Th2 responses. Indeed, laboratory mice maintained in barrier-free outdoor conditions mounted a Th1/Th2 mixed type response to whipworm infection and maintained high numbers of worms, whereas SPF mice responded with highly biased Th2 responses and thereby terminated the infection more quickly^59^. Hence, although type-2 cytokine- and antibody-mediated resistance to GI nematode infections typically rises with age in human and wildlife populations, it is conceivable that IFN-γ availability related to host age, genetic background and the infection history with other pathogens impacts this process in natural systems in similar ways as reported here for primary nematode infection under highly controlled environmental conditions.

## Methods

### Animals and infection

Female BALB/c and C57BL/6 mice were purchased from Janvier, Saint-Berthevin, France. Mice were house in specific-pathogen-free conditions in individual ventilated cages. Mice were infected by oral gavage of 200 3^rd^ stage larve of *H. polygyrus*. All experiments were compliant with German Animal Ethics Committee for the protection of animals (G0176/16, G0113/15).

### In vivo treatments

αIL-12p40 (C17.8, Biolegend) and αIFN-γ (XMG1.2, Biolegend) antibodies were administered at a dose of 0.5 mg intraperitoneally at days 0, 3 and 6 post infection. Recombinant IFN-γ (Peprotech) was given at a concentration of 2.5 μg in 200 μl DPBS intraperitoneally twice a day from day 0 to 4 post infection. Where possible, treated and control mice were housed together to reduce bias.

### Parasitological parameters

To determined fecal egg counts, weighed pellets collected from individual mice were briefly soaked in water (1 ml), meshed uniformly using a glass rod and mixed with saturated NaCl solution (6 ml) and counted using McMaster slides (FiBL). For the quantification of parasite fecundity, where possible, eight adult female worms were collected per mouse and cultured for 24 h at 37 °C and 5% CO_2_ in 200 µl of RPMI (200 U/ml penicillin and 200 μg/ml streptomycin, all from PAN-Biotech) on 96 well plates, followed by counting of the deposited eggs. To assess larval fitness by means of egress of the L4 stage from the infected tissue, a segment of 2–3 cm in length was cut from the proximal third of the freshly retrieved small intestine, followed by individual culture with both ends tied on 6 well plates in in serum free RPMI (PAN Biotech containing L-glutamine and 1% P/S). Larval egress was monitored for a total duration of 4 h and expressed as % of total L4 larvae present in each gut segment.

### Preparation of single cells

Single cells were prepared from spleen, mLN and small intestinal lamina propria (siLP) as previously described^11^. 1–2 drops of blood were collected in FACS buffer (PBS; PAN-Biotech with 0.2% BSA, 2 mM EDTA) and treated with FACS Lysing solution (BD Biosciences) to remove red blood cells before staining.

### Detection of parasite-specific CD4+ T cell responses

Bone marrow derived cells were harvested from tibia and femur of naïve BALB/c mice and cultured for 6 days with 20 ng/ml GM-CSF (Peprotech, Germany) to allow the differentiation of dendritic cells. Dendritic cells were pulsed with excretory/secretory products of *H. polygyrus* adult worms (HES, 5–10 µg/ml) for 5–6 h. Splenocytes were added to the pre-loaded dendritic cells and co-cultured overnight in the presence of Brefeldin A (Thermo Fisher, 3 µg/ml) and subsequently stained for surface and intracellular markers.

### Flow cytometry

For detection of intracellular cytokines, cells were stimulated with 1 μg/ml phorbol 12-myristate 13-acetate (Sigma-Aldrich) and 1 μg/ml ionomycin (Sigma-Aldrich) at 37 °C and 5% CO_2_ for a total of 4 h, brefeldin A (3 µg/ml) was added after 30 min. Cells were either fixed using IC fixation buffer or fix/perm buffer when targeting transcription factors (both from Thermofisher) and stained using the following reagents: CD4 (clone RM4-5; Alexa 700, Brilliant Violet 510, or PerCP), FoxP3 (clone FJK-16s; PE-eFluor610, eFluor 450, Alexa 488, or PerCP-Cy5.5), T-bet (clone 4B10; PE), GATA-3 (clone TWAJ; eFluor 660), IFN-γ (clone XMG1.2; eFluor 450, Alexa 700, or PE-eFluor610), IL-4 (clone BVD6-24G2; Biotin), IL-13 (clone eBio13A; Alexa 488), CD154 (clone MR1; Biotin), CD44 (clone IM7; PerCP), CD62-L (clone MEL-17; APC-eFluor780), RELM-α (Biotin), F4/80 (clone BM8; eFluor 450), Siglec-F (clone: E50-2440; PerCP-Cy5.5). Streptavidin coupled to PE, PE-Cy7 or APC was used as secondary conjugates. Non-specific binding of antibodies was prevented by adding FcγRII/III blocking antibody (clone 93). Dead cells were excluded using Fixable Viability Dye eFluor 780 or eFluor 506. All antibodies and other reagents were from BioLegend, Thermofisher, or BD Biosciences. Cells were acquired on FACSCanto^TM^ II (BD Biosciences) or FACSAria^TM^ III (BD Biosciences) and data analyzed using FlowJo (Tree star Inc., Ashland, USA).

### Statistics

All statistical analysis were performed using GraphPad Prism Software (San Diego, CA, USA). Normality was tested with the Shapiro–Wilk test, followed by ordinary one-way-ANOVA or Kruskal–Wallis test and Tukey’s or Dunn’s multiple comparison test. Comparisons of two groups were performed with an unpaired *t* test or Mann–Whitney test.

## Supplementary information

Supplementary Materials

## Data Availability

The raw data of the study are available upon request.
