# Supplementary material for: Age-dependent rise in IFN-γ competence undermines effective type 2 responses to nematode infection
Source: Mucosal Immunol. 2022 Jun 11;15(6):1270–82. doi: 10.1038/s41385-022-00519-6 (PMC9705248; doi:10.1038/s41385-022-00519-6)
Supplement: Supplementary file 1 — Supplementary Materials [file 41385_2022_519_MOESM1_ESM.pdf]

Fig. S1

Homeostatic expansion of IFN-γ competent cells with age

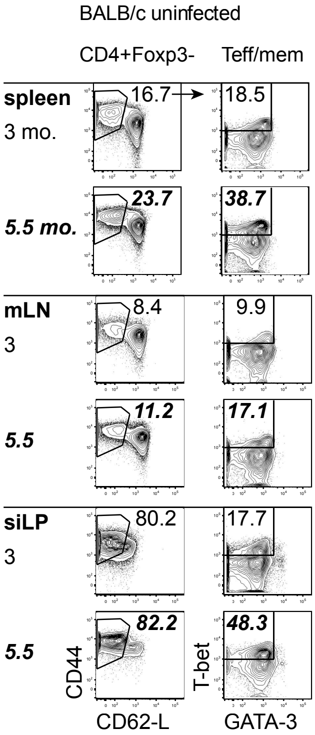

Fig. S1: Homeostatic expansion of IFN-γ competent cells with age. a Left: Representative contour plots depicting CD44 vs. CD62L expression in CD4+FoxP3- cells in spleen, mLN and small intestine in uninfected BALB/c mice. Right: Corresponding contour plots depicting the T-bet vs. GATA-3 expression in CD44+ CD62L- cells.

Fig. S2

Early type 2 adaptive responses in Hp infected adult and mature mice

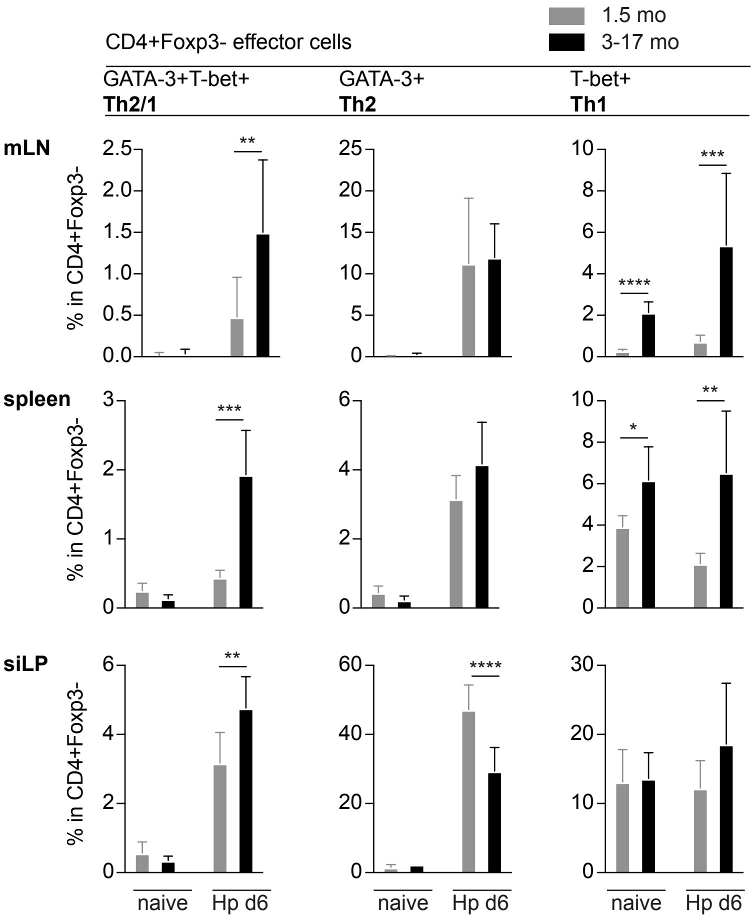

Fig. S2: Early type 2 adaptive responses in Hp infected adult and mature mice. Mature mice display an early impairment in Th2 responses. a Bar graphs depict the frequencies of Th2/1, Th2 and Th1 cells in top: mLN, mid: spleen and bottom: small intestine in CD4+FoxP3- cells in adult and mature BALB/c mice. Data from six independent experiments are reported (n=3-10 mice/group) as mean + SD. Statistical analysis was performed separately between adult and mature naïve mice and between adult and mature Hp infected mice using unpaired t test or Mann-Whitney test. *$p\leq0.05$, **$p\leq0.01$, ***$p\leq0.001$, ****$p\leq0.0001$.

Fig. S3

Susceptible C57BL/6 mice accumulate more Th2/1 cells compared to resistant BALB/c mice.

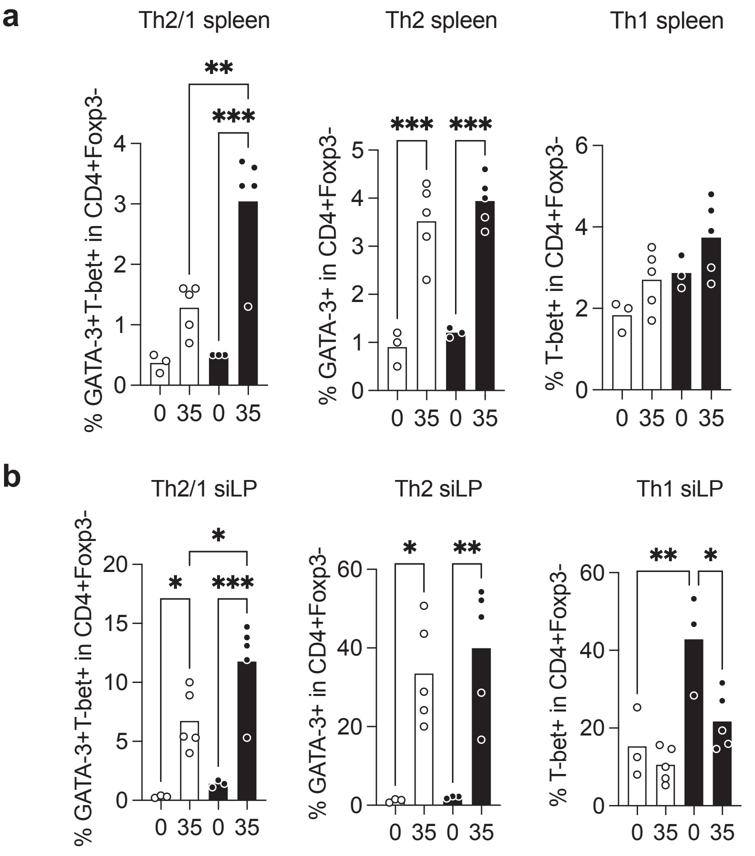

Fig. S3: Susceptible C57BL/6 mice accumulate more Th2/1 cells compared to resistant BALB/c mice. Graphs depict the requencies of Th2/1, Th2 and Th1 cells in spleen (**a**) and small intestine (**b**) at indicated time points post infection. Data derive from one of two experiments with similar results. Bar report the mean, circles represent individual mice. Statistical analysis was performed using one-way ANOVA and Tukey's multiple comparisons test or Kruskal Wallis test with Dunn's multiple comparisons test depending on the normality tests.*p≤0.05, **p≤0.01, ***p≤0.001.

Fig. S4

Highly resistant SJL mice accumulate fewer Th2/1 cells associated with higher M2 polarization

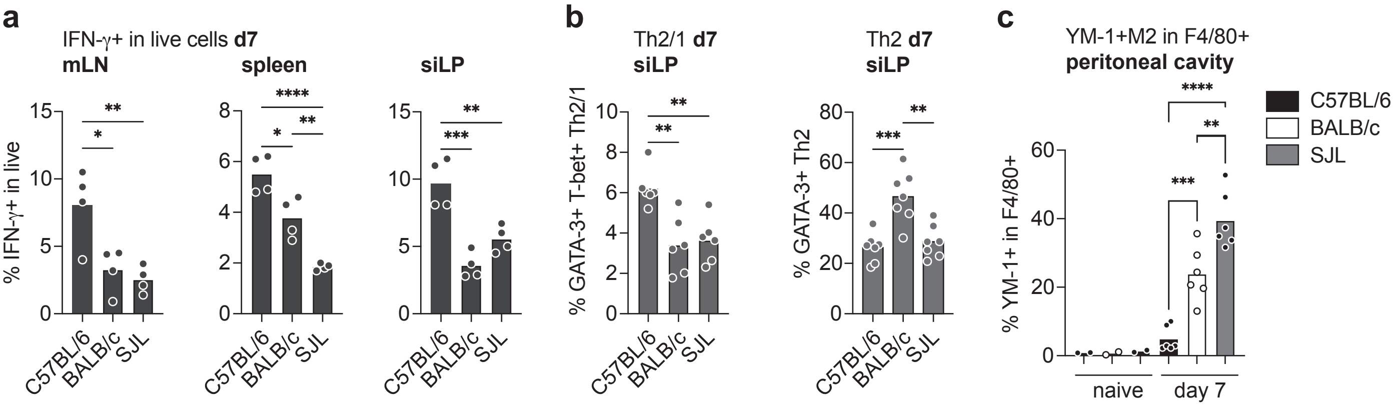

Fig. S4 Highly resistant SJL mice accumulate fewer Th2/1 cells associated with higher M2 polarization. a Frequencies of IFN-γ producing cells in mLN, spleen and siLP shown as percentage in live cells at day 7 post infection. Cells were stimulated with PMA/Ionomycin. Data derive from one out of two experiments with similar results (n=4 mice/group). **b** Frequencies of left: Th2/1 and right: Th2 cells in siLP at day 7 post infection. Cells were stained for transcription factors *ex vivo*. **c** M2 polarization depicted by the frequencies of YM-1+ cells in F4/80+ macrophages in peritoneal cavity in left: C57BL/6, mid: BALB/c and right: SJL mice. Data from two experiments each performed with n=4 mice/group are pooled, one sample/mouse line was used for other purposes (b, c). Bars report the mean, circles represent individual mice. Statistical analysis was performed using one-way ANOVA and Tukey's multiple comparisons test or Kruskal Wallis test with Dunn's multiple comparisons test depending on the normality tests. *p≤0.05, **p≤0.01, ***p≤0.001, ****p≤0.0001.
